# Protective Effects of Tiaoganquzhi Decoction in Treating inflammatory Injury of Nonalcoholic Fatty liver Disease by Promoting CGI-58 and Inhibiting Expression of NLRP3 Inflammasome

**DOI:** 10.3389/fphar.2022.851267

**Published:** 2022-05-02

**Authors:** Huicun Zhang, Xiang Gao, Pengmin Chen, Hongbing Wang

**Affiliations:** ^1^ Beijing Hospital of Traditional Chinese Medicine, Capital Medical University, Beijing, China; ^2^ Beijing Institute of Chinese Medicine, Beijing, China; ^3^ China-Japan Friendship Hospital, Beijing, China; ^4^ Beijing Hospital of Traditional Chinese Medicine Yanqing Hospital, Beijing, China

**Keywords:** Tiaoganquzhi decoction, non-alcoholic fatty liver disease, CGI-58, ROS, NLRP3, IL-1β

## Abstract

Tiaoganquzhi Decoction (TGQZD) is a traditional Chinese herbal formulation demonstrated to be a clinically effective treatment for nonalcoholic fatty liver disease (NAFLD), although details concerning its clinical mechanism are poor. This study aimed to explore the mechanism of TGQZD on improvement of inflammatory damage and dyslipidemia caused by NAFLD through the CGI-58/ROS/NLRP3 inflammasome pathway. In our research, the *in vivo* protective effects of TGQZD on HFD-induced liver injury in rats and *in vitro* using lipopolysaccharide (LPS)+palmitate (PA)-stimulated HepG-2 cells model. Histological changes were evaluated by hematoxylin-eosin and Oil Red O staining. Inflammatory cytokines and protein expression were analyzed by ELISA, Real time PCR and western blotting. Liver function, blood lipids, free fatty acids (FFA), and reactive oxygen species (ROS) were determined by biochemical detection. Our results indicated that TGQZD exhibited anti-inflammatory activity, reduced the severity of NAFLD and ameliorated the pathological changes. Further, TGQZD improved liver function and lipid metabolism in NAFLD rats. TGQZD lowered serum aspartate aminotransferase, alanine aminotransferase, triglyceride, and total cholesterol levels. TGQZD suppressed the formulation of FFA and ROS. It also reduced the expression and release of the inflammatory cytokine interleukin-1β by promoting CGI-58 expression and inhibiting the expression of FFA, TNF-α, and the NLRP3 inflammasome induced by ROS. TGQZD exhibited anti-inflammatory effects via the CGI-58, ROS and NLRP3 inflammasome pathway *in vivo* and *in vitro*, respectively. Our findings demonstrated that TGQZD is a useful and effective therapeutic agent for treating NAFLD via promotion of CGI-58 to inhibit the expression of ROS-induced NLRP3 inflammasome.

## Introduction

Nonalcoholic fatty liver disease (NAFLD) is the first cause of chronic liver disease, currently the prevalence of NAFLD in the world is 6.3–33% (Chalasani et al., 2012). NAFLD has become the most common chronic liver disease in the world. NAFLD is a clinicopathological syndrome that causes fatty degeneration of liver parenchyma cells due to excessive fat accumulation. NAFLD lesions generally develop from simple steatosis to steatohepatitis (NASH), liver fibrosis, cirrhosis and liver necrosis (Hasegawa et al., 2020), and may even transform into liver failure and liver cancer (Soresi et al., 2020). Therefore, it is of great significance to prevent and treat NAFLD.

NAFLD is a complex disease regulated by many factors and mechanisms, including environment, metabolism, gene, immunity and intestinal microecology, etc. Steatosis is a necessary condition for NAFLD. According to the classic theory of “second strike” in the pathogenesis of NAFLD, excessive accumulation of lipids in the cytoplasm of hepatic cell caused by various reasons are the first strike, which decreases the anti-strike ability of liver. The second attack is caused by the increase of reactive oxygen species (ROS), intestinal lipopolysaccharide (LPS), some cytokines secreted by immune system and adipose tissue, which triggered a series of cytotoxic events, leading to the inflammatory over reaction of liver (Day and James, 1998). The occurrence and progress of NAFLD mainly include fat accumulation in viscera, insulin and leptin resistance, oxygen stress, lipid peroxidation injury, inflammation of adipose tissue and liver tissue, etc. Inflammasome in liver cells may play an important role in the “second strike” response (Arab et al., 2018; Chen et al., 2020).

Comparative gene identification 58 (CGI-58) is named asα/βhydrolase domain-containing 5 (Abhd5) and expressed widely. Mutations CGI-58 in human will cause Chanarin-Dorfman syndrome (CDS) and it is a neutral lipid storage disease that the characterion is ichthyosis (thickened dry skin) and accumulation of triglyceride rich lipid droplets are found in most tissues and cell types (Lefevre et al., 2001). The role of CGI-58 in mediating intracellular fat hydrolysis is well established. CGI-58 has been shown to be involved in the intracellular fat hydrolysis (Lass et al., 2006; Brown et al., 2007; Brown et al., 2010; Zierler et al., 2013), but research concerning how CGI-58 is linked to inflammasome activation is limited.

The inflammatory corpuscle is a kind of multi protein complex which is involved in innate immune defense function and assembled by the cytoplasmic pattern recognition receptor (PRR), When cells are stimulated by external signals, specific inflammasome recruit and activate caspase-1, which processes and self activates and secretes cytokines IL-1β, IL-18 and TNF-αto enhance the inflammatory reaction of the body against internal and external stimuli (Lee et al., 2015; Sarkar et al., 2019). Nod-like receptor protein 3 (NLRP3) is the most well-known inflammasome. It is mainly composed of NLRP3, ASC (known as the apoptosis-associated speck-like granular protein with a CARD domain) and caspase-1 (a hydrolase containing hemitryptophan and hydrolysable aspartic acid protein). After receiving the activation signal, NLRP3 first combines with ASC, and ASC then recruits pro-caspase-1 to the multiprotein complex. The binding of multiple pro-caspase-1 clusters is unstable and is rapidly activated by via autocatalytic activity to produce active caspase-1 (Wan et al., 2016). Caspase-1 processes pro-IL-1β into IL-1β. IL-1β once released exerts effects outside the cell and activates a series of inflammatory chain reactions (Ajmera et al., 2017). NLRP3 inflammasome has been shown to be associated with a variety of immune diseases. At present, few studies have investigated the pathogenesis of NLRP3 inflammasome in NAFLD (Lee et al., 2015; Gao et al., 2016; Shao et al., 2018).

Specific CGI-58 knockout in mice can increase HFD-induced impaired glucose tolerance and insulin resistance (IR), which are associated with inflammation. CGI-58-deficiency leads to overproduction of ROS, which activates the NLRP3 inflammasome to secrete proinflammatory cytokines. CGI-58 as a suppressor inhibits the activation of the NLRP3 inflammasome in HFD-induced NAFLD mice (Miao et al., 2014).

Clinical studies have shown that the Tiaoganquzhi decoction (TGQZD) can improve the clinical symptoms of NAFLD patients, and has a certain role in regulating blood lipids and in reducing body weight. Compared with silibinin capsule, it has certain advantages in improving clinical symptoms, liver function and regulating blood lipids (Qi, 2012). However, there are few reports that TGQZD inhibited inflammatory damage of hepatocytes and improved the metabolism of lipid by regulated CGI-58 and the NLRP3 inflammasome. The aim of this study was to observe the effect of TGQZD on NAFLD rats and to explored whether its mechanism is related to the regulation of CGI-58 and NLRP3inflammasome expression, which will provide a reference for the treatment of NAFLD and deepen the therapeutic effect mechanism of TGQZD.

## Materials and Methods

### Laboratory Animals

Male Sprague-Dawley (SD) rats, weighting 120 ± 20 g, were obtained from Beijing Weitong Lihua Research Center for Experimental Animals. Rats were maintained in a temperature-controlled room (25 ± 1 °C in 12–12 h light-dark cycles) and housed in the animal facilities at the Beijing Hospital of Traditional Chinese Medicine (Capital Medical University Beijing, China). The study was carried out under the established guidelines for animal experimentation and the protocol was approved by the Animal Studies Ethics Committee of Beijing Hospital of Traditional Chinese Medicine, Capital Medical University with code number 2019030201.

### Preparation of TGQZD

TGQZD consists of the following 13 dried crude herbs: *Astragalus membranaceus* (Fisch.) Bge, *Bupleurum chinense* DC, *Atractylodes macrocephala* Koidz, *Curcuma wenyujin* Y. H. Chen et C. Ling, *Pinellia ternata* (Thunb.) Breit, *Artemisia scoparia* Waldst. etKit, *Alisma orientale* (Sam.) Juzep, *Cassia obtusifolia* L, *Salvia miltiorrhiza* Bge, *Angelica sinensis* (Oliv.) Diels, *Paeonia lactiflora* Pall, *Crataegus pinnatifida* Bge. var. Major N. E. Br and *Semen sinapis.* The ratio of the TGQZD formulation is 3:2:4:3:1:4:3:3:6:3:3:3:2 (Table 1). On the basis of standards specified in the Chinese Pharmacopoeia (2015 edition), Beijing Hospital of Traditional Chinese Medicine, Capital Medical University provided all the herbs for this preparation. The herbs were chopped into crude herbs and mixed. TGQZD was boiled in distilled water at 100°C for 2 h for extraction. Then the TGQZD solution was concentrated to the density of 2 g crude herb/ml and stored at −20 °C until further use.

**TABLE 1:** Different components in the formula of TGQZD.

Chinese name	Latin name	Used part	Traditional use	Voucher specimens
Huang qi	*Astragalus membranaceus* (Fisch.) Bge	root	Qi Reinforcing	TCM - 201101 - JPQCHSD01
Chai hu	*Bupleurum chinense* DC.	root	To relieve fever, to soothe the liver	TCM - 200926 - JPQCHSD02
Bai zhu	*Atractylodes macrocephala* Koidz	root	Qi-reinforcing	TCM - 200816 - JPQCHSD03
Yu jin	*Curcuma wenyujin* Y. H. Chen et C. Ling	tuberoid	Blood activating Stasis Removing	TCM - 201022 - JPQCHSD04
Ban xia	*Pinellia ternata* (Thunb.) Breit	tuber	Phlegresolving	TCM - 201007 - JPQCHSD05
Yin chen	*Artemisia scoparia* Waldst.etKit	aboveground part	Diuretic Dampness Excreting	TCM - 200901 - JPQCHSD06
Ze xie	*Alisma orientale* (Sam.) Juzep	tuber	Diuretic Dampness Excreting	TCM - 201019 - JPQCHSD07
Jue mingzi	*Cassia obtusifolia* L	ripe seed	Fire Purging	TCM - 201022 - JPQCHSD08
Danshen	*Salvia miltiorrhiza* Bge	root	Blood-activating and Stasis-removing	TCM - 200930 - JPQCHSD09
Dang gui	*Angelica sinensis* (Oliv.) Diels	root	Blood-Tonifying	TCM - 200903 - JPQCHSD10
Chi shao	*Paeonia lactiflora* Pall	root	Heat Clearing Blood Cooling	TCM - 201024 - JPQCHSD11
Shan zha	*Crataegus pinnatifida* Bge. var. Major N. E. Br	rosaceae	help digestion and Stasis-removing	TCM - 201009 - JPQCHSD12
Bai jiezi	*Semen sinapis*	ripe seed	removing the phlegm	TCM - 200526 - JPQCHSD13

### Ultra-high-performance Liquid Chromatography-Tandem Mass Spectrometry (UPLC-MS/MS) Analysis of TGQZD

Then the components of the TGQZD were analyzed by liquid chromatography/mass spectrometry (LC/MS)/MS instrument (Thermo Fisher). Briefly, DIONEX Ultimate 3,000 (Thermo Fisher) ultrahigh-performance liquid chromatography and Thermo Hypersil Gold C18 column (1.7 μm × 2.1 mm × 100 mm) were used to analyze sample. The mobile phase consisted of A (water, 2 mmoL/L ammonium formate, and 0.1% formic acid, v/v) and B (acetonitrile) with gradient elution. The gradient conditions for C18 separation was as follows: 100% A and 0% B, initial; 70% A and 30% B, 2 min; 30% A and 70% B, 9 min; 5% A and 95% B, 11 min; 0% A and 100% B, 12 min; 0% A and 100% B, 14 min; 100% A and 0% B, 14.1 min, 14 min; 100% A and 0% B, 16 min. The flow rate was 300 μl/min and the injection volume was 1 µl. The column temperature was maintained at 45°C. Mass spectrometry analysis was performed by the Q Exactive mass spectrometer (Thermo Fisher). The voltage of positive and negative ion source were 3.7 and 3.5 kv, respectively, and the heated vaporizer temperature was maintained at 320°C. Data were collected and processed by the Xcalibur 2.2 SP1.48 software (Thermo Fisher).

### *In vivo* Experimental Design

Eighteen 6-week-old male Sprague-Dawley rats were randomly divided into three groups of six rats each. One group (normal diet, ND, n = 6) of rats was fed with 11.4% kcal fat diet (Beijing Science and Cooperation Feed Technology Limited Company, Beijing, China; protein: 27.5%, carbohydrate: 65.8%, and fat: 11.4% kcal/g), and the other two groups (high-fat diet HFD, n = 6), or a high-fat diet along with TGQZD, HFD + TGQZD, n = 6) were fed with a 33.1% kcal fat diet (Beijing Science and Cooperation Feed Technology Limited Company, Beijing, China; protein: 19.6%, carbohydrate: 47.1%, and fat: 33.1% kcal/g). The high fat diet was administered for 8 weeks to establish the NAFLD rat model (Al-Shaaibi et al., 2016; Liu et al., 2016). From the ninth week rats were dosed by oral gavage once per day for 8 weeks with TGQZD 5 ml/kg per day. The rats were treated for 8 weeks.

### Sample Collection

After 8 weeks of treatment, the rats were anesthetized by 10% pentobarbital sodium. After deep anesthesia abdominal blood samples from all rats were collected for serum biochemical assays. In order to minimize the time that rats suffered pain from experiment as much as possible, they were rapidly euthanized by cervical dislocation during deep anesthesia and then the liver was removed and washed in PBS before being placed in 4% paraformaldehyde. Liquid nitrogen was used to freeze the remaining liver tissue, which was stored at −80°C for subsequent analysis.

### Histological Examination and Assessment

Sections of liver samples (4-μm thick) were stained with hematoxylin-eosin (H&E), while frozen liver tissues (5-μm thick) were dyed with Oil Red O stain. Liver samples were examined under a light microscope (Olympus Medical Systems Corp, Tokyo, Japan). Severity was assessed by the NAFLD score. The evaluation system consisted of the following: steatosis (on a scale of 0–3), lobular inflammation (on a scale of 0–3), and hepatocellular ballooning (on a scale of 0–2). Higher scores indicated more serious disease (Kleiner et al., 2005).

### Serum Biochemical Parameter Analysis

The serum levels of alanine aminotransferase (ALT); aspartate aminotransferase dd (AST); blood triglyceride (TG), and total cholesterol (TC) were measured using the 7,160 Automatic Biochemical Analyzer (Hitachi, Japan) following the manufacturer’s operating instructions.

### Determination of FFA and ROS Levels in Liver

Liver tissues stored at −80 °C were then transferred to a homogenizer to obtain a tissue homogenate and then centrifuged at 3,500 rpm for 10 min to obtain the supernatant. The FFA and ROS levels in the supernatant were determined using the commercial kits (Nanjing Jiancheng Biochemical Co., Ltd. and Beijing Sino-Uk institute of Biological Technology, respectively).

### Enzyme-Linked Immunosorbent Assay

The serum levels of IL-1β and TNF-α were detected by enzyme-linked immunosorbent assay (ELISA) according to the manufacturers’ protocols (Beijing SINO-UK Institute of Biological Technology, cat. no. HY-10101, HY-H0019). Briefly, solid-phase antibodies are produced by microplates using purified biotin-based antibody packs, which are added to the colon tissue lysate and affinity labeled by ’spicy root peroxidase. After thorough washing, add 3, -3′, -5, -5′-tetamiphenyl benzene to develop color. The absorbance was measured at 450 nm and the sample concentration was calculated.

### Cell Culture and Treatment

The human hepatocellular carcinoma cell line (HepG-2) was purchased from cell resource center of Shanghai Institutes for Biological Sciences Chinese Academy of Sciences. HepG-2 cells were cultured in DMEM containing 10% fetal bovine serum and maintained in 6-cm dishes under a humidified atmosphere with 5% CO_2_ at 37°C in a cell culture incubator. When cells reached 60% confluence, the cells were divided into four groups: the Control, TGQZD, Lipopolysaccharide (LPS) + palmitate (PA), and LPS + PA + TGQZD groups. In the Control group, cells were treated with phosphate-buffered saline (PBS) in the culture medium; TGQZD group was treated with TGQZD in the culture medium; the LPS + PA group was treated with 10 μg/ml LPS+0.4 mM PA in the culture medium and the LPS + PA + TGQZD group was treated with 4 mg/ml TGQZD and 10 μg/ml LPS+0.4 mM PA in the culture medium, each group of cells was incubated at 37 °C with 5% CO_2_. After 24 h of the co-culture, cells in each treatment group were collected and analyzed as described below.

The Institute of Traditional Chinese Medicine, Chinese Academy of Traditional Chinese Medicine provided TGQZD lyophilized powder. 100 mg TGQZD lyophilized powder were dissolved in 5 ml DMEM. The drug solution is sterilized after filtration through the filter membrane and stored at 4°C. TGQZD-dried powder solution was diluted to the required concentration prior to experimentation.

### Cell Viability Assays

Cell viability was determined using MTT. HepG-2 cells were seeded at 1 × 10^4^ cells/well in 96-well plates and then incubated with MTT for 4 h at 37°C. Absorbance was measured at 490 nm and the optical density (OD) values were compared to determine whether the TGQZD have an effect on cell viability.

### Measurement of Lipid Accumulation and Intracellular ROS Formation

HepG-2 cells were seeded at 2 × 10^5^ cells/well in a 6-well plate. When HepG-2 cells reached 70% confluence, cells were treated with 0, 4 mg/ml TGQZD and LPS + PA mixture for 24 h, respectively. The TG levels were measured using a commercial kit according to the manufacturer’s instructions (Applygen Technologies Inc. Beijing, China). A fluorescence probe (DCF-DA) was used to detect the ROS levels of HepG-2 cells according to the manufacturer’s instructions (Beijing Sino-Uk institute of Biological Technology).

### RT-PCR

RNA was extracted from the liver tissue and HepG-2 cells were lysed in ice-cold samples preserved at −80 °C using a kit according to the manufacturer’s protocol. The following PCR primers were synthesized. NLRP3-Human: forward, 5ʹ-CCACAAGATCGTGAGAAAACCC-3ʹ and reverse, 5ʹ-CGG​TCC​TAT​GTG​CTC​GTC​A-3ʹ; CGI-58-Human:forward,5ʹ-CACATGGTGCCCTACGTCTAT-3ʹ and reverse, 5ʹ-ACAGGT​CTG​TTG​GTG​CAA​AGA-3ʹ; ACTIN-Human:forward, 5ʹ-CCT​GGC​ACC​CAG​CAC​AAT-3ʹ and reverse, 5ʹ-GGG​CCG​GAC​TCG​TCA​TAC-3ʹ; NLRP3-Rat: forward, 5ʹ-TGC​ATG​CCG​TAT​CTG​GTT​GT-3ʹ and reverse, 5ʹ-AGC​TGA​GCA​AGC​TAA​AGG​CT; CGI-58-Rat: forward, 5ʹ-CGGCAGTGATGAAAGCGATG-3ʹ and reverse, 5ʹ-TAG​ACG​TGG​GAC​ACC​AGG​TA; ACTIN-Rat:forward, 5ʹ-TCC​ACC​CGC​GAG​TAC​AAC​C-3ʹ and reverse, 5ʹ-CGA​CGA​GCG​CAG​CGA​TA (Sangon Biotech, Shanghai, China). cDNA was synthesized using a RevertAid first-strand DNA synthesis kit (Tiangen Biotech). ABI-7500 Real-time quantitave PCR thermal cycl and a real-time PCR system kit (Tiangen Biotech) were used to detect Thermal cycler gene expression according to the manufacturer’s instructions and cycle threshold (CT) values were calculated by normalizing CT to ACTIN expression to present target gene (2-ΔΔCT) expression. The SYBR Green real-time PCR cycling protocol used was as follows: DNA denaturation at 95°C for 3 min, followed by 40 cycles at 95°C for 5 s and 60°C for 33 s (Zhang et al., 2021).

### Western Blot Analysis

The liver tissue and HepG-2 cells were lysed in ice-cold RIPA lysis buffer (Beyotime, Shanghai, China) for 30 min. Protein extracted were centrifuged at 20000r/min×10 min at 4 °C. BCA method (Beyotime Biotechnology, China) was used to determine the lysate protein concentration. The supernatant with buffer solution was then boiled at 100°C for 10 min and stored at −20 °C.

10% SDS gel electrophoresis (SDS-PAGE) was used to separate 60 µg liver and 30 µg HepG-2 cells denatured proteins solution. Then the protein in the gel was transferred to the nitrocellulose membrane. The membrane was sealed in TBST with 8% nonfat milk for 1 h at 37°C, then CGI-58 (sc-100468) antibody, NLRP3 antibody (AF2155) and anti-ACTIN (AA128) were added and incubated overnight at 4°C. After washing the membrane in TBST for three times for 10 min each, the secondary antibody was added and incubated at 37°C for 1 h. The film was washed in TBST for three times for 10 min each again, and was scaned by an Odyssey scanner (LI-COR). Then image was analyzed by ImageJ software.

### Statistical Analysis

The statistical analyses was performed using statistical software package SPSS 17.0 and data were reported as means ± standard deviation. The normality test was performed by Shapiro-Wilk test, and then the differences among multiple groups were analyzed by one-way analysis of variance (ANOVA). ANOVA was followed by the least significant difference (LSD) post hoc test for pairwise comparison. A *p* value less than 0.05 was considered statically significant.

## Results

### Identification of the Components of the TGQZD Formula Using UPLC-MS/MS

**FIGURE 1:**
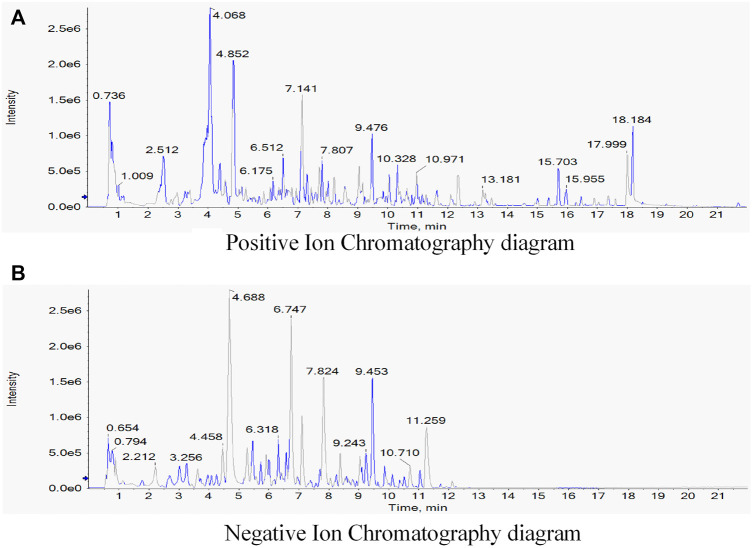
Identification of components of Tiaoganquzhi Decoction(TGQZD) using UPLC-MS/MS. The positive **(A)** and negative **(B)** ion chromatograms of TGQZD were shown as indicated. The major component and their retention time were listed in the (Table 2).

The composition of TGQZD were analyzed by UPLC-MS/MS. The total ion chromatogram of the TGQZD is shown in Figure 1. According to the Chinese pharmacopeia (2015), the components identified in TGQZD were listed as follows: ferulic acid, salvigenin, saikosaponin A, bisdemethoxycurcumin, angelicoidenol, 1,2-dihydrotanshinone, astragaloside II, astragaloside I, galloylpaeoniflorin, paeonidaninol A, astraverrucin IV, paeonin A, saikosaponin C, salvianolic acid B, aurantio-obtusin, chrysophanol, atractylenolide I, and alisol B 23-acetate. They were eluted at 4.3, 6, 10.01, 10.3, 8.46, 12.24, 9.81, 10.32, 5.73, 8.03, 8.03, 8.26, 9.28, 6.76, 9.23, 12.33, 10.55 and 12.79 min, respectively (Table 2).

**TABLE 2:** The components identified in TGQZD.

Compound	m.z	Retention.time.min
Ferulic acid	193.0504	4.3
Salvigenin	373.0929	6
Saikosaponin A	825.4658	10.01
Bisdemethoxycurcumin	307.0972	10.3
Angelicoidenol	215.1292	8.46
1,2-Dihydrotanshinone	277.087	12.24
Astragaloside II	871.4727	9.81
Astragaloside I	867.4766	10.32
Galloylpaeoniflorin	631.1676	5.73
Paeonidaninol A	629.1879	8.03
Astraverrucin IV	843.4763	8.03
Paeonin A	629.1882	8.26
Saikosaponin C	971.5254	9.28
Salvianolic acid B	717.148	6.76
Aurantio-Obtusin	329.0673	9.23
Chrysophanol	255.0651	12.33
Atractylenolide I	231.1379	10.55
Alisol B 23-acetate	515.3718	12.79

### TGQZD Attenuates HFD-Induced TG Accumulation

**FIGURE 2:**
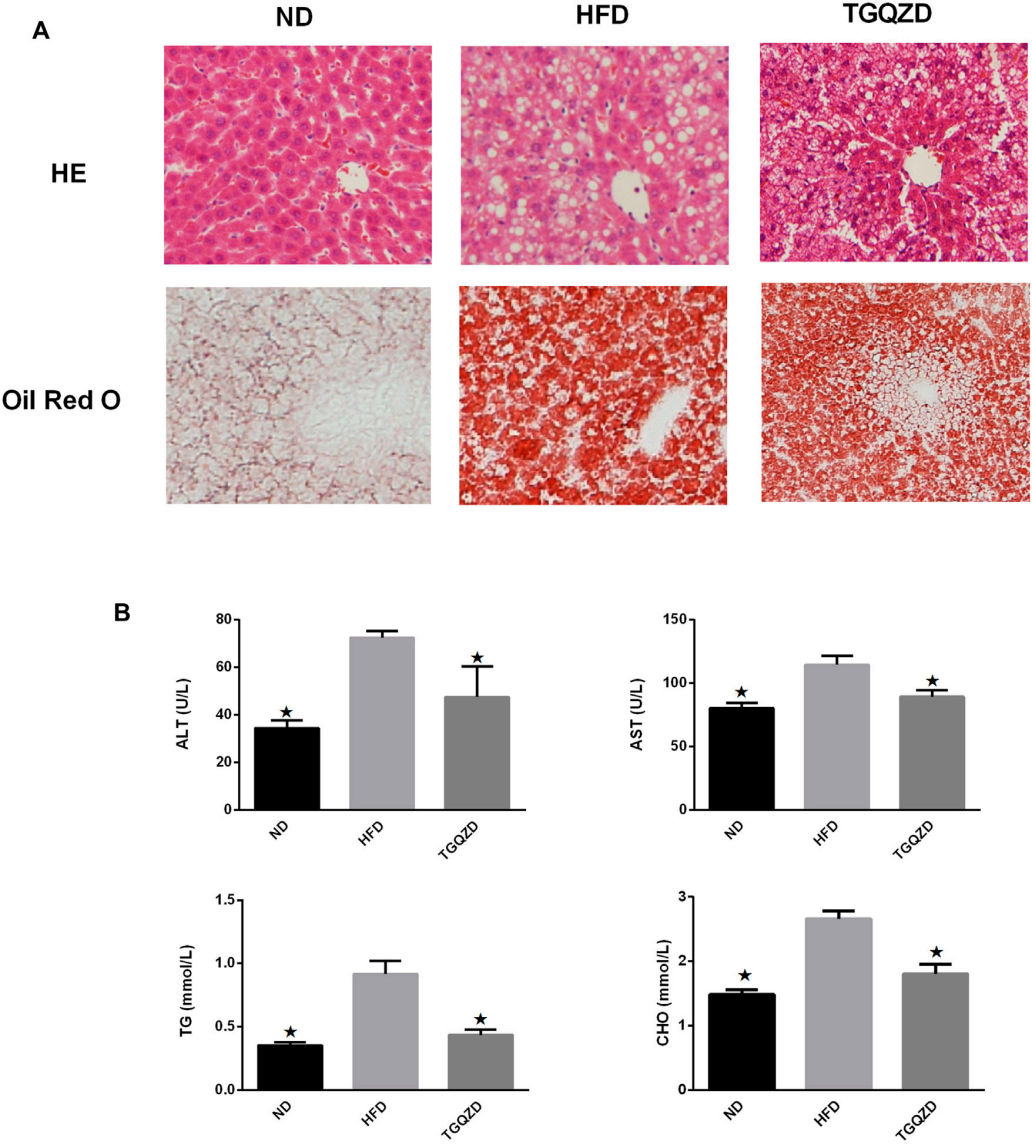
**(A)**Effect of TGQZD on liver histomorphology of NAFLD rats. The HE staining showed that the morphology and structure of the liver tissue in the ND group were normal; In the HFD group, fatty degeneration appeared in the liver. Hepatocytes are swollen and round and their volume is significantly higher than that of ND group. There exist a large number of fat vacuoles and balloons in cytoplasm. After the treatment of TGQZD, the fatty vacuoles and balloon like changes in NAFLD rats’ liver were improved. The results of oil red O staining showed that there was little red lipid drop in the liver tissue of the ND group and a large number of red lipid drops in the liver tissue of the HFD group, which showed that the lipid droplets infiltrated into the hepatocytes and fused into pieces. Red lipid droplets were also found in liver tissue of rats in TGQZD group, but the number and the fusion into pieces were less than that in HFD group (Figure 2). Histological examination showed that fatty degeneration, inflammation, and hepatocyte ballooning in TGQZD group were alleviated than HFD group (Table 3). **(B)** Effect of TGQZD on liver function and blood lipid of NAFLD rats. Compared with the ND group, the levels of ALT, AST, TG and TC in the HFD group were significantly higher (*p* < 0.05); compared with the HFD group, the levels of ALT, AST, TG and TC in the TGQZD group were significantly lower (*p* < 0.05). *p < vs. HFD Group, alone.

H&E staining revealed that the morphology and structure of the liver tissue. There were limited red lipid droplets in the ND-treated group. In the HFD group, fatty degeneration appeared in the liver. Hepatocytes were swollen and round and their volume was significantly larger than that of the ND group. A large number of fat vacuoles and evidence of balloon degeneration was detected in the cytoplasm. Following treatment with TGQZD, the fatty vacuoles and balloon-like changes in the livers of NAFLD rats improved. Consistent with the H&E staining, the results of Oil Red O staining showed that a large number of red lipid droplets in the liver tissue of the HFD group. While lipid droplets in the TGQZD group were less than that in the HFD group (Figure 2A). Histological examination showed fatty degeneration, inflammation, and hepatocyte ballooning in the TGQZD group were alleviated compared with the HFD group (Table 3).

**TABLE 3:** Average score of histopathological findings in livers ( $\bar{x}\pm s$ ,*n = 6*).

Group	Steatosis	Inflammation	Ballooning
ND	0	0	0
HFD	2.00 ± 0.00^*^	2.46 ± 0.03^*^	2.00 ± 0.00^*^
TGQZD	1.33 ± 0.03^**^	1.23 ± 0.03^**^	1.30 ± 0.05^**^

Quantitative data are expressed as mean ± SD., Statistical analysis of the data for multiple comparisons was performed by one-way ANOVA.*p<0.05**,** versus the ND, group, **p<0.05**,** versus the HFD, group.

Compared with the ND group, the levels of ALT, AST, TG, and TC levels in the HFD group were significantly higher (*p* < 0.05); compared with the HFD group, the levels of ALT, AST, TG, and TC in the TGQZD group were significantly lower (*p* < 0.05) (Figure 2B).

### TGQZD Lowered FFA and ROS Levels in Liver of NAFLD Rats

**FIGURE 3:**
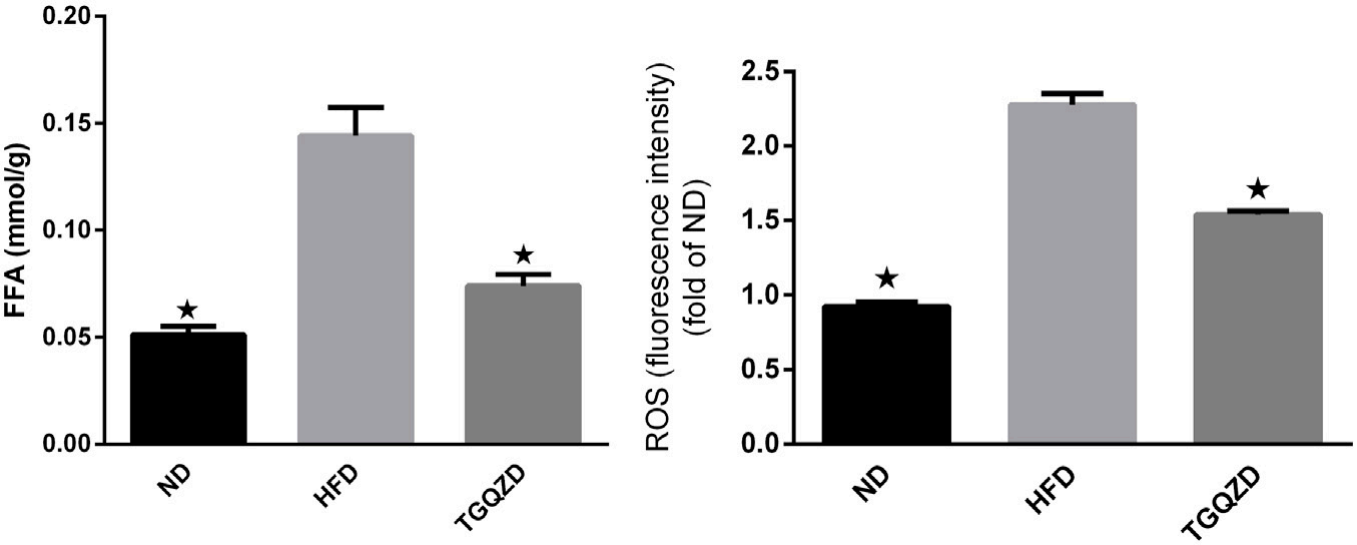
Effect of TGQZD on FFA and ROS content in liver of NAFLD rats. Compared with the ND group, the content of FFA and ROS in the liver of NAFLD rats was significantly increased (*p* < 0.05), while compared with the HFD group, TGQZD significantly reduced the level of FFA and ROS in the liver of NAFLD rats (*p* < 0.05). *p < 0.05 vs. HFD Group, alone.

Compared with the ND group, FFA and ROS levels in the HFD group were significantly higher (*p* < 0.05), while Compared with the HFD group, TGQZD had significantly lowered levels of FFA and ROS in NAFLD rats (*p* < 0.05) (Figure 3).

### TGQZD Decreased Serum IL-1β and TNF-α Content in NAFLD Rats

**FIGURE 4:**
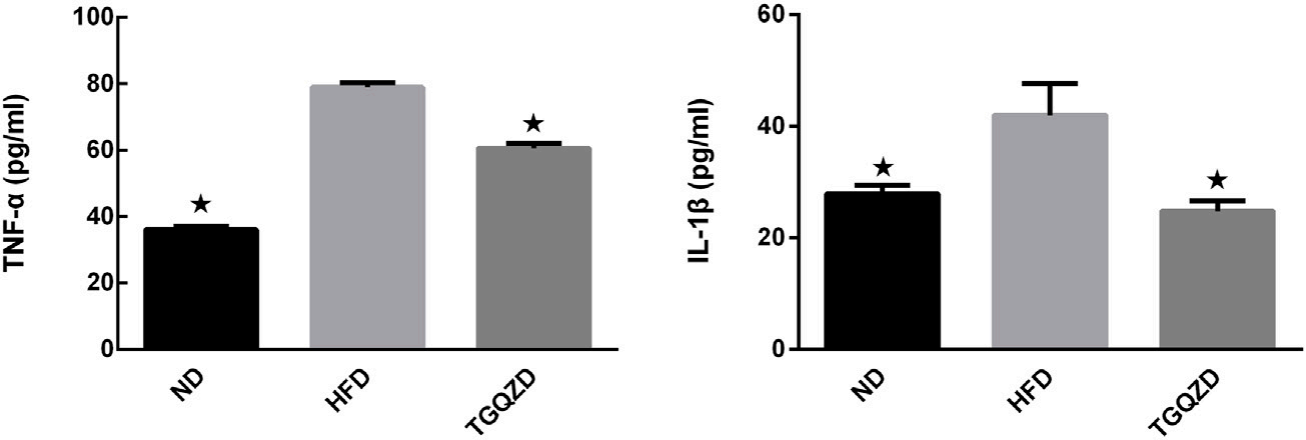
Effect of TGQZD on serum IL-1 β content in NAFLD rats. After 8 weeks of treatment, compared with the ND group, the level of serum IL-1β in HFD group increased significantly (*p* < 0.05); while compared with the HFD group, the level of serum IL-1β in the TGQZD group decreased significantly (*p* < 0.05), indicating that TGQZD could effectively inhibit the formation of pro-inflammatory cytokines by reducing the increased level of IL-1 β in NAFLD rats. *p < 0.05 vs. HFD Group, alone.

Compared with the ND group, the levels of serum IL-1β and TNF-α in HFD group increased significantly (*p* < 0.05). Compared with the HFD group, the levels of serum IL-1β and TNF-α in the TGQZD group decreased significantly (*p* < 0.05) (Figure 4).

### TGQZD Improved the Viability of Hepatocytes-Stimulated With LPS + PA

**FIGURE 5:**
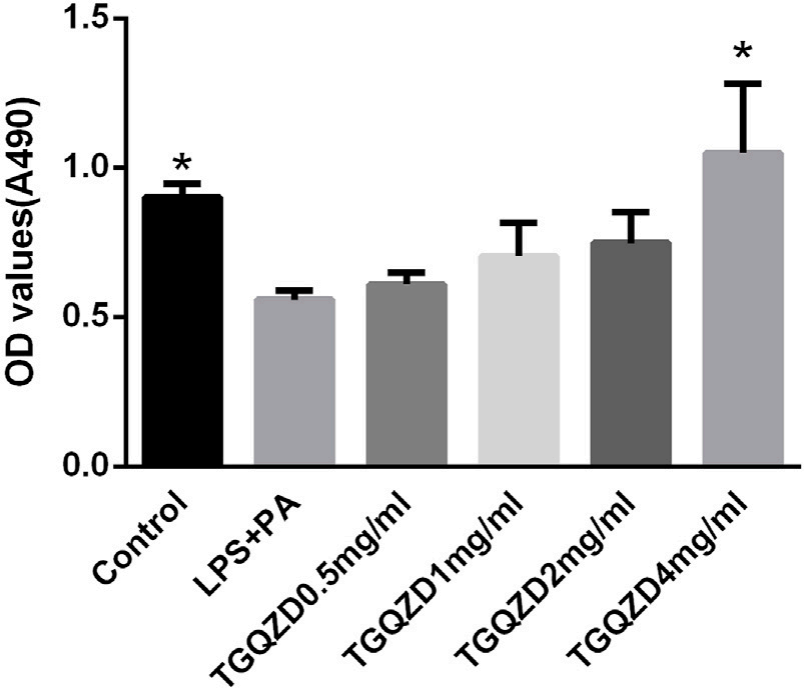
Effects of TGQZD on the viability of HepG-2 cells. HepG-2 cells were treated with 10ug/ml LPS+0.4 mM palmitate and different concentrations of TGQZD for 24 h, and cell viability was measured using the MTT assay. Data are expressed as the mean ± SD (n = 3). *p < 0.05 vs. LPS + PA Group, alone.

After being stimulated with LPS + PA for 24 h, there was a significant difference in the rate of cell proliferation between in the LPS + PA and Control groups. Compared with the Control group, the cell proliferation of the LPS + PA group was markedly decreased. Compared with the LPS + PA group, TGQZD treatment resulted in a significant increase in cellular proliferation (Figure 5).

### TGQZD Reduced Cellular TG and ROS Levels in HepG-2 Cells Exposed to LPS and PA

**FIGURE 6:**
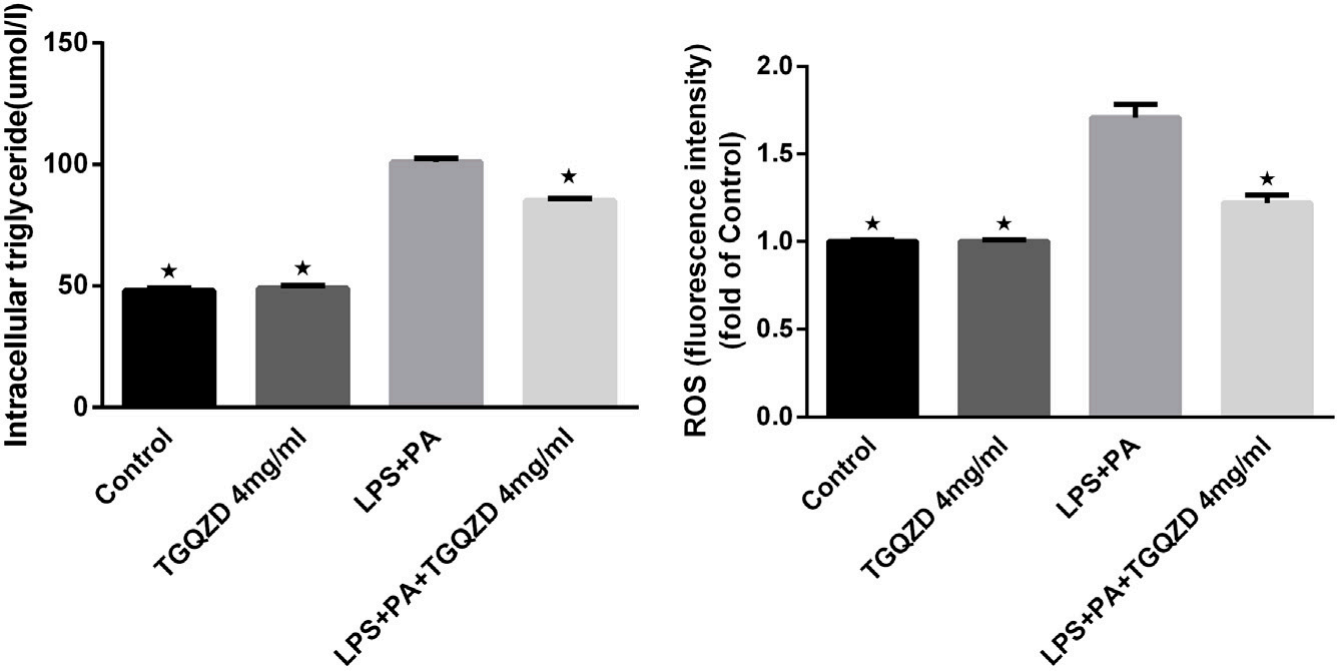
Effect of water extract TGQZD on the formation of intracellular triglyceride (TG) and reactive oxygen species (ROS) in LPS + PA -treated HepG-2 cells. In the Control group, phosphate-buffered saline (PBS) in the culture medium; TGQZD group was treated with TGQZD in the culture medium; the LPS + PA group was treated with 10 μg/ml LPS + 0.4 mMPA in the culture medium and the LPS + PA + TGQZD group was treated with 4 mg/ml TGQZD and 10 μg/ml LPS + 0.4 mMPA in the culture medium, respectively. *p < 0.05 vs. LPS + PA Group, alone.

Following a 24 h treatment of HepG-2 cells with LPS + PA, the TG concentrations and ROS levels in the LPS + PA group were obviously higher than in control group. Meanwhile, TG and ROS production in the LPS + PA + TGQZD treatment group were markedly decreased compared with the LPS + PA group (*p* < 0.05). Compared with the control group, there were no significant TG and ROS change in the TGQZD alone group (Figure 6).

### TGQZD Increased CGI-58 Expression and Reduced NLRP3 Inflammasome in HepG-2 Cells Stimulated With LPS and PA

**FIGURE 7:**
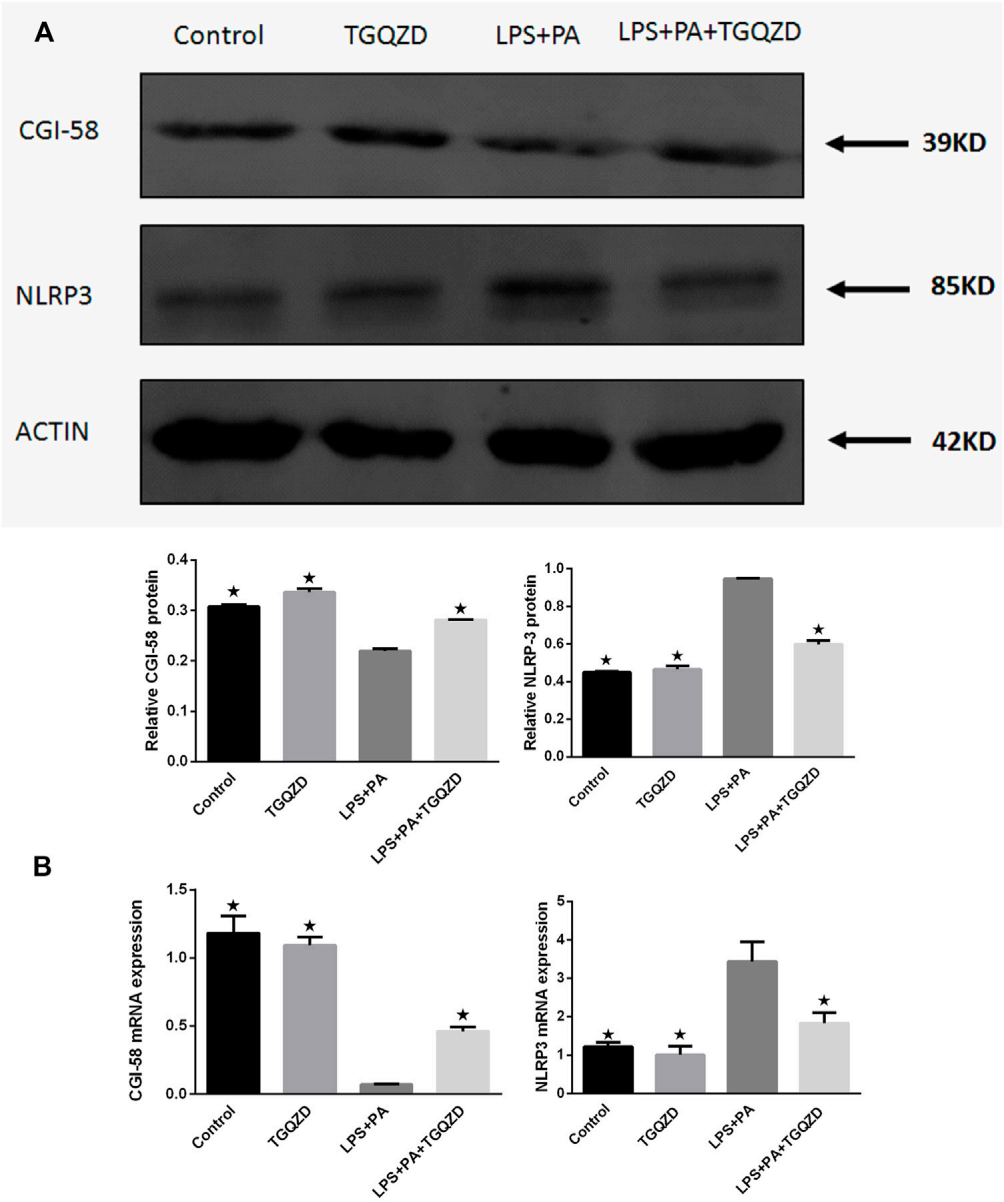
Effect of TGQZD on CGI-58 and NLRP3 inflammasome of HepG-2 cells. **(A)** Representative images of Western blot showing CGI-58 and NLRP3 inflammasome protein expression in HepG-2 cells in the different groups. **(B)** Quantitation of CGI-58 and NLRP3 inflammasome mRNA expression in HepG-2 cells in different groups. ACTIN was used as a loading control. *p < 0.05 vs. LPS + PA Group, alone, (*n* = 3).

Real time PCR and western blot analysis indicated, in HepG-2 cells stimulated with LPS + PA for 24 h, the expression of CGI-58 in the LPS + PA group was inhibited, while CGI-58 increased in the LPS + PA + TGQZD group. Compared with the control group, the expression of NLRP3 inflammasome in the LPS + PA group was overexpressed. TGQZD decreased the overexpression of NLRP3 inflammasome induced by LPS + PA. Compared with the control group, there were no significant CGI-58 and NLRP3 inflammasome change in the TGQZD alone group (Figure 7).

### TGQZD Promoted CGI-58 Expression and Inhibited NLRP3 Inflammasome Formation in the Liver of NAFLD Rats

**FIGURE 8:**
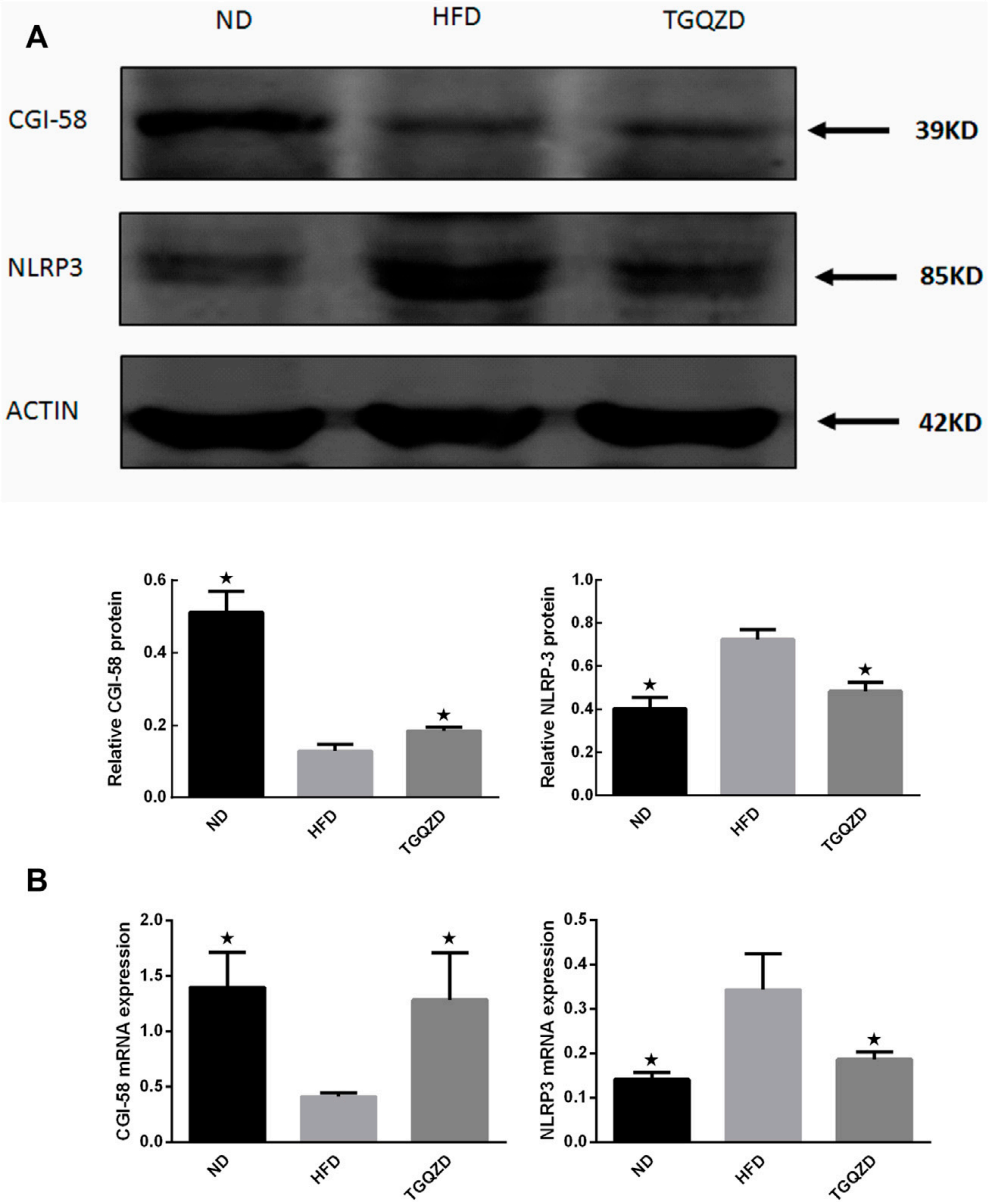
Effect of TGQZD on CGI-58 and NLRP3 inflammasome of liver in NAFLD rats. **(A)** Representative images of Western blot showing CGI-58 and NLRP3 inflammasome protein expression in NAFLD rats in the different groups. **(B)** Quantitation of CGI-58 and NLRP3 inflammasome mRNA expression in NAFLD rats in different groups. ACTIN was used as a loading control. *p < 0.05 vs. HFD Group, alone, (*n* = 3).

After TGQZD treatment, compared with the ND group, the hepatic levels of CGI-58 decreased, while NLRP3 inflammasome in the HFD group increased significantly (*p* < 0.05). Compared with those of the HFD group, TGQZD significantly promoted the formation of CGI-58 and inhibited the expression of NLRP3 inflammasome (*p* < 0.05) (Figure 8).

## Discussion

As with earlier research, SD rats fed by the high-fat diet were developed NAFLD asssocited with hepatic glycerol accumulation and hyperlipidaemia (Marchesini et al., 2003). In our study, the HFD fed rats have significant biochemical features of NAFLD, including significantly elevated liver enzymes and hyperlipidemia accompanied by an increase in the accumulation of triglyceride in the liver. The histological abnormalities in the HFD rats of this study were consistent with the findings of the previous literatures (Kim et al., 2017).

Researchers have widely used animal model of NAFLD induced by high-fat diet to identify the pathogenesis and explore its treatment for NAFLD (Barbuio et al., 2007).

The nonspecific clinical feature of NAFLD is elevated hepatic aminotransferase (ALT and AST) and they are positively correlated with most patients with NAFLD (Bacon et al., 1994). After Treatment with TGQZD, elevated hepatic aminotransferase were significantly reduced. The results of biochemical and histological analysis showed that TGQZD have a protective effect on HFD-induced liver damage.

The pathogenesis of NAFLD has not been clarified. Abnormal fat metabolism, oxygen stress, and lipid peroxidation, heredity, hormones, the microenvironment, and drugs may all play important roles in the excessive fat accumulation in the liver. Obesity and IR increases the levels of FFAs and promotes the accumulation of TGs in the liver as the first strike (Li et al., 2018) and changes the permeability of membrane. It promotes the release of inflammatory factors, swelling, inflammation, and necrosis. The second attack was mainly caused by the self-injury of intracellular environmental factors including ROS, lipid peroxidation products, inflammasome, and inflammatory reactions caused by downstream inflammatory factors (Gao et al., 2020). Oxidative stress causes the peroxidation of membrane phospholipid and changes the permeability of the membrane. It promotes the release of inflammatory factors. Swelling, inflammation, and necrosis of hepatocytes lead to liver fibrosis, which can develop into cirrhosis (Canada et al., 2019). The liver is a key organ of fat metabolism. It is an important site of fat digestion, absorption, oxidation, decomposition, transformation, and maintenance of the balance of lipid metabolism in the body. If too much fat is ingested, the level of blood lipids is increased, thus the fat entering the liver exceeds the threshold of liver transformation, TG in hepatocytes are increased, apolipoproteins are insufficient, and the release of TGs from the liver is reduced, which leads to a large amount of TGs accumulating in the liver, eventually causing NAFLD (Grasselli et al., 2019). Clinically, NAFLD is often accompanied by damage in liver function and disorder in liver lipid metabolism. In the present study, compared with the HFD group, the levels of ALT, AST, TG, and TC in the TGQZD group were significantly lower (*p* < 0.05), which indicated that TGQZD could reduce the damage due to the presence of high fat levels in hepatocytes and improve the lipid metabolism of NAFLD rats. An analysis of the pathological sections also showed that TGQZD alleviated the liver damage, lipid deposition, and fatty degeneration of hepatocytes in NAFLD rats. Nonetheless, the abnormal fat metabolism, the inflammatory response by hepatocytes, the oxygen stress may play an important role in the formation and development of NAFLD (Yamada and Guo, 2018).

When the liver cell membrane is damaged, an inflammatory response is triggered, reducing various functions of liver, and causing excessive fatty accumulation in liver (Teimouri et al., 2020). The mitochondria are the main sites of fatty acid β-oxidation and trifusoic acid cycle. Lipid peroxidation leads to the formation of free radicals, which can directly damage the structure of cell membrane and brings about its functional disorder (Wang et al., 2020). Free radicals can react with the double bonds of fatty acyl lipids of the cell membrane to generate MDA. MDA affects mitochondrial activity and blocks fatty acid oxidation, which results in fat accumulation in the liver (Ji et al., 2019). Excessive lipid peroxide also can induce inflammatory cell infiltration, activate Kupffer’s cell, and HSC, which can cause liver fibrosis (Liang et al., 2018). We showed that TGQZD effectively reduced the levels of FFA and ROS in NAFLD rats. This indicated that TGQZD could improve lipid metabolism and reduce the level of oxidative stress in NAFLD rats.

CGI-58 is a key regulator of TG conversion. CGI-58 regulates the mobilization of TG by stimulating adipose TG lipase (ATGL) enzyme activity. In neutral lipid storage disease patients, the presence of CGI-58 mutation is more strongly associated with the potential development of fatty liver disease and hepatomegaly than the ATGL mutation (Lord and Brown, 2012), which indicates that CGI-58 has an independent function of ATGL in the liver. CGI-58 gene knockout leads to hepatic steatosis, CGI-58 regulates the storage and secretion of liver neutral lipids in the absence of the ATGL gene. The regulation of hepatic triacylglycerol metabolism by CGI-58 does not require ATGL co-activation (Lord et al., 2016). CGI-58 is not only a lipolytic factor, but it is also an endogenous inhibitor of NLRP3 inflammasome activity. CGI-58 deficiency induced ROS accumulation and then activated NLRP3 inflammasome, which caused liver inflammatory injury induced by HFD in mice (Miao et al., 2014). In our study, TGQZD might promote the formation of CGI-58 and inhibit the expression of NLRP3 inflammasome, which can effectively improve the lipid metabolism disorder and inflammatory injury of hepatocytes induced by HFD.

ROS are key mediators in the activation of caspase-1 and the NLRP3 inflammasome (Dostert et al., 2008; Zhou et al., 2011). Studies have shown that mitochondrial dysfunction activates the NLRP3 inflammasome by inducing ROS accumulation (Wen et al., 2011). NLRP3 combines with ASC to activate caspase-1, and then activates IL-1β, which promotes the release of inflammatory factors, and thus aggravates the inflammatory response (Tilg et al., 2016). IL-1β could promote hepatocytes to take up fatty acids, synthesize TG, and aggravate the steatosis of liver. Under the condition of a high-fat diet, IL-1ra deficient mice exhibited a more severe fatty hepatitis than wild-type mice (Matsuki et al., 2003). These studies indicated that IL-1β could promote the accumulation of lipids and the development of inflammation in the liver (Stojsavljevic et al., 2014; Ajmera et al., 2017). The generation of ROS also led to the activation of the transcription factor NF-κB, which in turn leads to an increase in the production of TNF-α (Syn et al., 2009). TNF-α levels were found to be elevated in NASH patients (Wigg et al., 2001). Further, serum TNF-α levels have been associated with NAFLD and may be involved in hepatocellular inflammation and IR in NAFLD (Valenti et al., 2002). TGQZD could effectively inhibit the formation of pro-inflammatory cytokines by reducing the upregulation of IL-1β and TNF-α levels in NAFLD rats. Thus, regulating CGI-58 and NLRP3 inflammasome are of great significance for the treatment of NAFLD in the future.

The “multiple parallel strikes” process emphasizes the parallel effects of different injury factors (Tilg and Moschen, 2010). The combined interaction of PA and LPS with toll-like receptors (TLRs) can increase NLRP3 inflammasome and pro-IL-1β expression (Wan et al., 2016). PA can be absorbed by the scavenger receptor CD36, which as a TLR activator and endophage agent, will promote the activation of NLRP3 inflammasome (Kagan and Horng, 2013). In order to study further mechanism of improving NAFLD fat metabolism *in vitro*, We applied PA and LPS as “parallel hits” to mimic NASH environment. HepG-2 cells, were incubated with LPS + PA and TGQZD. It is similar to the results of animal experiments, TGQZD decreased the NLRP3 inflammasome and ROS level while increased CGI-58 expression in HepG-2 cells induced by LPS + PA.

**FIGURE 9:**
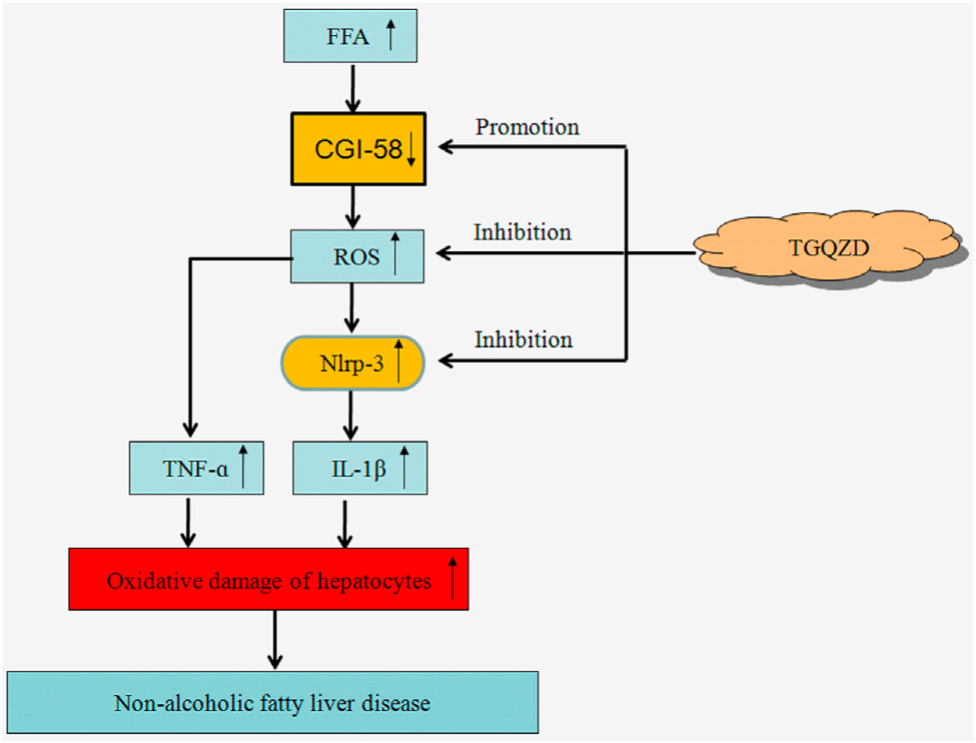
The therapeutic effect of TGQZD on NAFLD rats. Based on the above, TGQZD can reduce the accumulation of lipid in liver by regulating the level of lipoproteins; improved hepatic function; inhibit FFA and promote CGI-58 expression to suppress activating the ROS-dependent NLRP3 inflammasome pathway, which reduce the liver inflammation injury.

As discussed above, regulating CGI-58 and NLRP3 inflammasome will be of great significance for the treatment of NAFLD in the future. In this study we found that after the treatment with TGQZD, the CGI-58 levels increased significantly, while NLRP3 inflammasome protein, IL-1β, and TNF-α levels decreased significantly, which also suggested that TGQZD exerted certain protective effects on NAFLD-induced rats through the regulation of CGI-58 and NLRP3 inflammasome (Figure 9).

TGQZD significantly improved the liver function; blood lipids; the body weight of NAFLD patients (Qi, 2012). According to the dose conversion between rats and human (Nair and Jacob, 2016), we didn’t design different doses of TGQZD in the experiment. TGQZD is prepared from the aqueous extracts of 13 medicinal herbs and thus may contain many different chemical compounds.

It has been reported that ferulic acid can ameliorate hepatic inflammation and modulate specific gut microbiota and genes involved in TG and TC metabolism (Ma et al., 2019; Wu et al., 2021). Salvigenin improve the metabolic syndrome symptoms by decreasing lipid levels and stimulating mitochondrial functionality and it also help cells to survive by inhibiting apoptosis and enhancing autophagy (Rafatian et al., 2012; Serino et al., 2021). Saikosaponin A, modulate glycerolipid metabolism role by regulating Lipe and Lipg and transcription factors peroxisome proliferator-activated receptor alpha (PPARa) (Li et al., 2021).

With the help of UPLC-MS/MS analysis of TGQZD, ferulic acid, salvigenin and saikosaponin A were also found in TGQZD. Combining previous research with our research data, TGQZD exerts its therapeutic effect may not only through regulation of CGI-58 and NLRP3 inflammasome, but also modulation the above targets and their related signal pathways, which needs to be confirmed in our follow-up study. It also refects that TGQZD posess the advantage of multi-target in the treatment of NAFLD.

Due to NAFLD belong to a complex systemic metabolic disease, it is not easy to design the ideal drugs for single target. Multi target therapy or combination therapy have recently become the main treatment to reduce the symptoms of NAFLD (Corey and Chalasani, 2011; Johnston et al., 2020), which is consistent with multi-target of TGQZD for treatment NAFLD.

To clarify the exact therapeutic effect of TGQZD in the treatment of NAFLD, we will perform a follow-up study to identify the main active ingredients of TGQZD that are responsible for the hepatic protective activities, which will be the focus of our future studies.

## Conclusion

Our findings provide evidence that TGQZD can exert therapeutic effects on NAFLD and reduces the accumulation of lipids in the liver by regulating the level of lipoproteins; improving hepatic function, inhibiting FFA metabolism, and promoting CGI-58 expression to suppress the activation of the ROS-dependent NLRP3 inflammasome pathway, which ultimately reduces liver inflammatory injury. By inhibiting the formation of FFA and ROS formation, TGQZD induces CGI-58 expression, inhibits the formation of NLRP3 inflammasome, and TGQZD also could suppress the inflammatory response induced by IL-1 β and TNF-α.

## Data Availability

The original contributions presented in the study are included in the article/Supplementary Material, further inquiries can be directed to the corresponding author.
